# From vaccine to visa apartheid, how anti-Blackness persists in global health

**DOI:** 10.1371/journal.pgph.0001663

**Published:** 2023-02-27

**Authors:** Catherine Kyobutungi, Githinji Gitahi, Marie-Claire Wangari, Patterson Siema, Evelyn Gitau, Florence Sipalla, Madhukar Pai, Samuel Oji Oti

**Affiliations:** 1 African Population and Health Research Center, Nairobi, Kenya; 2 Amref Health Africa, Nairobi, Kenya; 3 Women in Global Health-Kenya Chapter, Nairobi, Kenya; 4 School of Population and Global Health, McGill University, Montreal, Canada; 5 Global Health Decolonisation Movement in Africa, Nairobi, Kenya; PLOS: Public Library of Science, UNITED STATES

Global health evolved from colonial medicine and hence deeply rooted in the white supremacy mindset [1]. Anti-Blackness is an inescapable consequence. Definitions of anti-Blackness revolve around the positioning of Black people, their cultural practices and knowledge as inferior, the conscious and unconscious dehumanization and discrimination of Black bodies, a disdain for Black people and their lived experiences, the disenfranchisement of Black people, but above all, a system of beliefs and practices that erode their humanity.

In a recent event held in Nairobi, Kenya, we discussed what anti-Blackness in global health means, why it matters, and what needs to be done to counter anti-Blackness in global health and development [2].

## How does anti-Blackness manifest itself?

No continent has been more impacted by the ravages of colonialism and racism than the African continent. Sadly, even today, Africans are at the receiving end of discrimination, from vaccine apartheid to visa apartheid.

The Covid-19 pandemic offers a stunning recent example of anti-Blackness. No continent is less vaccinated and boosted than the African continent [3]. While wealthy nations rushed to clean up the shelves, hoard vaccines, and even throw away millions of expired vaccines, the African region was left last in the line. Despite the efforts of activists and the support of most countries, a few rich countries blocked the TRIPS waiver that could have significantly expanded vaccine manufacturing in the Global South. Two years after vaccination began in wealthy nations, and even as second and third booster shots are being offered in the Global North, barely one in four people in the African region are vaccinated with two doses (as of January 2023) [3]. The African region has also had the lowest Covid-19 testing rate, and access to anti-viral medications such as Paxlovid is practically non-existent.

This pattern of discrimination is not new. More than 30 years ago, when anti-retrovirals (ARV) became available, they were considered too expensive to roll-out in the African region. As late as 2001, some experts maintained that ARV treatment in Sub-Saharan Africa was impossible. It took incredible activism, legal action, and community effort before they started becoming available, by which time millions of Africans got infected and died.

When the Ebola outbreak ravaged West Africa during 2014–16, it killed more than 11,000 people in Guinea, Liberia, and Sierra Leone. Even intravenous hydration was seen as being too challenging during this crisis. While an overwhelming majority of the mostly White American and European healthcare workers who contracted Ebola survived, the infection killed two-thirds of West Africans with Ebola. Investments in R&D dramatically increased only after Ebola infections began to be reported outside Africa; in fact, investment for new product development increased more than 900-fold after the outbreak in 2014 [4].

Africa is the only continent where mpox has been endemic for decades. And yet, when the global outbreak occurred, the west was prioritized for vaccine rollout. A giant share of the mpox vaccines is still held by the US, UK, Canada and France–some of the richest nations in the world, while the African region was once again left behind [5]. In the same vein, although sub-Saharan Africa carries the highest burden of cervical cancer cases and deaths globally, access to human papillomavirus vaccines remains very low in most African countries.

When the same patterns echo across diseases and across decades, it is hard to call it anything but anti-Blackness.

## Anti-Blackness is structural and dangerous

The de-prioritization of African lives by the rest of the world has harmful and deadly consequences. For example, in the sub-Saharan African countries most affected, HIV/AIDS lowered life expectancy among adults by about twenty years. Earlier access to ARVs could have prevented this devastating impact. Given the low vaccine and booster coverage in Africa against Covid-19, and emerging, highly immune-evasive subvariants, the African continent is poorly prepared to face any new, deadly variant of SARS-CoV2 as well as the consequences of long Covid.

Why is anti-Blackness so widespread? Historically, and even today, every aspect of global health is dominated by rich nations in the Global North. The health of Black people in Africa was never a priority for countries that colonized and exploited the continent; rather global health and its precursors grew out of the need to protect colonialists from diseases prevalent in colonised regions of the world. In fact, the act of colonial conquest was so violently disruptive that it radically altered lives and habitats leading to what was referred to in the early 1900s as the “pathological revolution” [6]. Hence, historically, decisions about the health of Black people were made in countries far away and not always in their best interests.

Are things better today? Sadly, data show we have a very long way to go. In 2020, less than 10% of the largest global health organizations were head-quartered in the African region, and very few of the global health organizations are led by African nationals [7]. A survey of over 2000 Board seats of global health organizations shows that less than 3% of the seats are held by nationals of low-income countries [8].

Vast amounts of global health funding are granted to the same global health organizations, even when the research or programmatic work is meant to be done in the African region. Even for diseases such as malaria, where Africa accounts for over 90% of malaria deaths, funding is often allocated to organizations in rich nations [9]. African researchers are often neither first nor senior authors on publications, even when the research is entirely done in Africa [10]. A survey of 615 journal editorial boards showed that less than 3% editors’ institutions were based in Africa [11].

When it comes to participation in international conferences and meetings, African delegates are often the least successful in obtaining visas, or have to work the hardest to even apply for them [12]. The recent examples of conferences in Montreal, Berlin, and New York are powerful examples of how challenging it is for African nationals to travel, share their work, study, work, or get recognized. The fact that African delegates have struggled to attend recent meetings in other Global South regions (e.g. Bogota, Colombia) is stark reminder that anti-Blackness is not confined to Global North countries. The harsh reality is that Africans continue to be largely spectators in a global health system that is paternalistic and strips them of agency and dignity even as it purports to serve them.

## What is the way forward?

Given the pervasive anti-Blackness, it is time to push back against the dominant ways of centering global health on people and countries with the most power and privilege. It is time for Africans to claim the seat they have historically been denied at the global health decision-making table [13]. It is time to abandon the charity model of global health, and demand for a model that is rooted in equity, human rights, justice, and self-determination.

African nations must work together in solidarity and realize the agenda of self-determination and self-reliance. As John Nkengasong, former head of Africa CDC, said, “Never ever should we have had to keep counting on externalities to take care of our own security needs. A key pathway for collective global security is an Africa that is self-sufficient [14].”

Indeed, Africa’s vision of a *New Public Health Order*, recently endorsed by African heads of state, is actively tackling health challenges and planning for the future, shaped by local leadership and regional solutions [15]. To create a new public health order, Africa will need to strengthen public health institutions, strengthen the health workforce, expand local manufacturing of products, increase domestic resources for health, and build respectful, action-oriented, and sustainable partnerships that promote country ownership and African health priorities.

To push back against the conference inequity, African experts may need to refuse to participate in global events where they are likely to be denied visas or treated with hostility. As the Yoruba saying goes, “you cannot shave a man’s head in his absence.” While advocating for a more just and equitable global health system, Africa must also develop its own platforms, which allow all Africans to attend without facing visa and passport discrimination. Indeed, such African conferences have already emerged, including the International Conference on Public Health in Africa (CPHIA) and Africa Health Agenda International Conference (AHAIC). At the same time, Africa must remove its own visa and other barriers to collaboration among people on the continent and harmonize frameworks that support seamless cross-border research and travel.

What does this vision for a new order in Africa mean for partners in high-income nations? As President Paul Kagame said ‘‘by doing things for ourselves, that does not mean acting alone.” For people with power and privilege, authentic allyship is a path forward (not white saviorism). People and organizations in the Global North must humbly listen to African experts, cede space, power and resources, and be supportive of the vision for a new public health order in Africa. At a minimum, partners in high-income nations must not obstruct this vision for self-determination.

In the end, Africa’s struggle for self-determination and against anti-Blackness will not be easily won. Like all big social justice movements, the fight will have to be won; it will not be conceded by those who benefit most from the status quo.
